# Ameliorative Effects of Boswellic Acid on Fipronil-Induced Toxicity: Antioxidant State, Apoptotic Markers, and Testicular Steroidogenic Expression in Male Rats

**DOI:** 10.3390/ani11051302

**Published:** 2021-04-30

**Authors:** Hossam G. Tohamy, Sara E. El-Kazaz, Saqer S. Alotaibi, Hawary S. Ibrahiem, Mustafa Shukry, Mahmoud A. O. Dawood

**Affiliations:** 1 Department of Pathology, Faculty of Veterinary Medicine, Alexandria University, Alexandria 22758, Egypt; hossam.gafar@yahoo.com; 2 Animals and Poultry Behavior and Management, Department of Animal Husbandry and Animal Wealth Development, Faculty of Veterinary Medicine, Alexandria University, Alexandria 22758, Egypt; sara@agr.kfs.edu.eg; 3 Department of Biotechnology, College of Science, Taif University, P.O. Box 11099, Taif 21944, Saudi Arabia; saqer@tu.edu.sa; 4 Department of Veterinary Pharmacology, Faculty of Veterinary Medicine, Alexandria University, Alexandria 22758, Egypt; hawary@agr.kfs.edu.eg; 5 Department of Physiology, Faculty of Veterinary Medicine, Kafrelsheikh University, Kafrelsheikh 33516, Egypt; 6 Department of Animal Production, Faculty of Agriculture, Kafrelsheikh University, Kafrelsheikh 33516, Egypt

**Keywords:** fipronil, boswellic acid, PCNA, semen, fertility-related markers

## Abstract

**Simple Summary:**

Fipronil (FPN) is an insecticide that can be used in insect control in various cereal crops in agriculture, veterinary activities, and public health management. Boswellic acid (BA) is a pentacyclic triterpene, which is a compound isolated from Boswellia serrata gum resin. This study was designed to determine BA’s potential protective impact against oxidative and testicular damage caused by FPN insecticide poisoning on the male rat model. BA significantly improved the reproductive parameters assessed, such as the number of pregnant females, index of pregnancy and the number of litters, weights of the reproductive organ, sperm cell quality, morphological alterations of testes, epididymis, and sex glands by accessory caused by FPN oxidative stress, as well as the improvement of steroidogenesis, antioxidants, and antiapoptotic marker.

**Abstract:**

The study investigated the ability of boswellic acid (BA) to alleviate the testicular and oxidative injury FPN insecticide intoxication in the male rat model. Rats were randomly assigned to six equivalent groups (six rats each) as the following: control rats orally administered with 2 mL physiological saline/kg of body weight (bwt); boswellic acid (BA1) rats orally administered 250 mg BA/kg bwt; boswellic acid (BA2) rats orally administered 500 mg BA/kg bwt; fipronil (FPN) rats orally administered 20 mg FPN/kg bwt; (FPN + BA1) rats orally administered 20 mg FPN/kg bwt plus 250 mg BA/kg bwt, and (FPN + BA2) rats orally administered 20 mg FPN/kg bwt plus 500 mg BA/kg bwt. After 60 days, semen viability percentage and live spermatozoa percentage were decreased, and a considerably increased abnormality of the sperm cells in FPN-administered rats improved substantially with the co-administration of BA. BA had refinement of the histological architecture of testes and sexual glands. Quantitative analysis recorded a noticeable decline in the nuclear cell-proliferating antigen (PCNA) percentage area. FPN triggered cell damage, which was suggested by elevated malondialdehyde and interleukin 6, tumor necrosis factors alpha, and decreased glutathione level. Proapoptotic factor overexpression is mediated by FPN administration, while it decreased the antiapoptotic protein expression. Similarly, BA has shown significant upregulation in steroidogenic and fertility-related gene expression concerning the FPN group. Pathophysiological damages induced by FPN could be alleviated by BA’s antioxidant ability and antiapoptotic factor alongside the upregulation of steroidogenic and fertility-related genes and regimented the detrimental effects of FPN on antioxidant and pro-inflammatory biomarkers.

## 1. Introduction

Fipronil (FPN) is an N-phenyl-pyrazole insecticide with a wide range and can be used in insect control in various cereal crops in agriculture, veterinary activities, and public health management [1]. FPN is an insect neurotoxin agent, and the critical mechanism is active when the GABA-regulated chloride channels are blocked, causing depression and death in the central nervous system [2]. FPN insecticides are the only organic toxicants to be applied to the ecosystem in a targeted manner to improve food safety by battling pests and regulating disease vectors [3]. Insecticide toxicity is well known to have multiple consequences; creating oxidative injury is a high concept due to reactive oxygen species [4]. Extensive use of these insecticides in agriculture and residential settings causes chronic neurological syndromes, teratogenicity, male reproductive failure, fetal growth retardation, embryo fetotoxicity, and genotoxicity [5]. Beyond liver toxicity, FPN also had reproductive effects, as studies found that the application of FPN influences fertility [6]. The excess output of reactive oxygen species leads to oxidative stress that decreases sperm fertility [7]. The level of serum hepatic enzymes and renal function biomarkers (creatinine and urea), cholesterol, and lactate dehydrogenase increased markedly in FPN intoxication; however, overall serum protein, albumin, and triglycerides decreased significantly, as well as a major increase in malondialdehyde and nitric oxide levels with a significant reduction in glutathione (GSH), glutathione peroxidase (GPx), superoxide dismutase (SOD), and catalase (CAT) [8]. FPN induced deterioration within the seminiferous tubules and apoptosis in the epididymides. Upregulated *interleukin-1β*, *nitric oxide synthase 2*, *caspase-3* (*Casp3*) as well as downregulated *Burkitt-cell lymphomas*, inhibin *B proteins*, and *androgen receptor* mRNA expressions *Casp3*, *nitric oxide synthase*, *ionized calcium-binding adapter molecule 1*, and *IL-1β* immunoreactions were increased. There was also a reduction of *proliferating cell nuclear antigen* (*PCNA*), *mouse vasa homolog* (*MVH*), and *SOX9* protein reactions [9].

Many studies report the effect of fipronil on the reproductive system on different animals in which Ohi et al. [10] reported that when fipronil was topically utilized to rats (single dose) at different concentrations (70, 140, and 280 mg/kg), it altered the cyclicity of female rats and had harmful reproductive effects in female rats. Mazzo et al. [6] reported that male rats that received fipronil 5 mg/kg for 14 days had decreased sperm production, reduced epidydimal sperm count, a reduction in GSH, and an increase in the concentration of malondialdehyde. In addition, Eisa et al. [5] reported that rats treated with different doses of fipronil 1/10 LD50 (2.1 mg/kg bwt) and 1/30 LD50 (0.7 mg/kg bwt) at the 6th to 15th days of pregnancy lead to teratogenic and embryotoxic effects. De Barros et al. [11] reported that pregnant rats exposed (via gavage) to fipronil (0.03, 0.3, or 3 mg/kg) from gestational day 15 until postnatal day 7 had infertility. In addition, Kitulagodage et al. [12] breeding female zebra finches orally dosed with single sublethal levels of fipronil (1, 5, and 10 mg/kg body weight) had a decrease in hatchability percentage.

Medicinal herbs are used extensively because of their antimicrobial, antioxidant, and less toxic effects than chemical substances. Boswellic acid (BA) is a pentacyclic triterpene, which is a compound isolated from Boswellia serrata gum resin. It is potent against several inflammatory diseases, including cancer, arthritis, ulcerative colitis, respiratory inflammatory disorders, brain tumor, fertility, and memory [13]. This gum-resin is applied in conventional Chinese medicine to remedy many aspects of well-being [14]. Many in vitro trials indicated that BA hinders the synthesis of the pro-inflammatory enzyme such as *5-lipoxygenase* (*5-LO*), *cyclooxygenase-1* (*COX-1*), *human elastase of the leucocytes* (*HLE*), *cathepsin G* (*cat G*), and *microsomal prostaglandin E* (*mPGES-1*), together with *nuclear factor kappa B* (*NF-μB*) inhibition and various cytokines such as *TNFα*, *IL-1β*, and *IL-6*, respectively [15]. Many clinical reports have also illustrated BA’s possible value as an anti-inflammatory agent [16,17,18] and antioxidant activity [19]. Therefore, this study was designed to determine BA’s potential protective impact against oxidative and testicular damage caused by FPN insecticide poisoning on the male rat model.

## 2. Materials and Methods

### 2.1. Animals and Management

Thirty-six healthy male albino rats (with a weight of 150 ± 10 g, ten weeks of age) were purchased from the Medical Research Institute, Alexandria University, Egypt. Rats were kept in separate plastic cages under unique conditions (23 ± 2 °C, 55% RH, and 12-h light/dark cycle) and had regular food and water ad libitum. The rats were acclimatized 14 days before the commencement of the experiment to restore normal behavior and growth. During the investigation, they were held under the same hygiene and environmental conditions. The research protocol was accepted by the Institutional Animal Care and Use Committee, Faculty of Veterinary Medicine, Alexandria University, and it was precisely designed under the consideration of animal welfare (AU013202062958).

### 2.2. Chemicals and Reagents

Fipronil (FPN) was purchased as a commercial product (Rado-X 80%WG, Cruz Agro Development and Investment, Ltd., Shanghai, China), and boswellic acid (BA) has also been procured as hard gelatin capsules (Boswellia serrata dry powder extract 500 mg) (Atos Pharma Phyto Pharmaceuticals Company, Cairo, Egypt). Biochemical kits have been purchased from Biodiagnostics Co. (Cairo, Egypt).

### 2.3. Experimental Design

**Figure 1:**
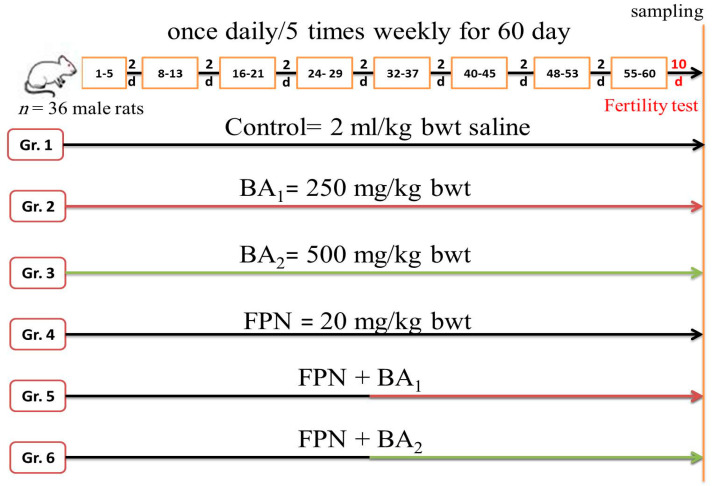
The experimental design. FBN, Fipronil. BA1, Boswellic Acid 250 mg/kg bwt. BA2, Boswellic Acid 500 mg/kg bwt. Gr, Group.

Rats were randomly assigned to six equivalent groups (six rats/each) as follows: Group 1 (control) rats were orally administered with two ml physiological saline/kg body weight (bwt) (a vehicle for other drugs); Group 2 (BA1) rats were orally administered 250 mg BA/kg bwt; Group 3 (BA2) rats were orally administered 500 mg BA/kg bwt according to Nusier et al. [20]. The utilized doses of boswellic acid in the current study were tested previously as showed by Sami et al. [21] and Nusier et al. [20] as well as Barakat et al. [22] and Tawfik et al. [23]. Al-Yahya et al. [24] reported that the boswellic acid is safe up to 1000 mg/kg in rats. Still, this dose is relatively high, considering the amount of extract consumed by humans. So, in this study, we try to investigate the effect of boswellic acid in ameliorating the toxic impact of fipronil in a dose-response manner. Group 4 (FPN) rats were orally administered 20 mg FPN/kg bwt, which corresponded to one-fifth of the LD50 [25]; Group 5 (FPN + BA1) rats were orally administered 20 mg FPN/kg bwt plus 250 mg BA/kg bwt, and Group 6 (FPN + BA2) rats were orally administered 20 mg FPN/kg bwt plus 500 mg BA/kg bwt. All treatments were given by stomach tube once daily/five times weekly for 60 days. The co-treatment group with BA was administered an hour before FPN administration. The FPN concentrations were determined by using FPN commercial formulation depending on the percentage of the active ingredient. Concentrations of FPN and BA were freshly made, and body weights were checked weekly throughout the experiment. Ten days after the last dose, the rats have been euthanized and the sample was collected. The experimental designs are shown in Figure 1.

### 2.4. Fertility Test

Adult male rats were introduced to porous untreated females in the ratio 1:2. Animals were left together for ten days, during which two estrous cycles should have elapsed. Female rats were injected intraperitoneally with LUTALYSE^®^ (dinoprost tromethamine) 0.1 mg/100 g bwt twice per day in the morning and late afternoon to synchronize estrous. Then, vaginal smear was performed to check for the presence of estrous. Females in estrous were introduced to each male present in the plastic cage individually. Vaginal smears were collected daily and examined under a microscope, and every positive female was followed up until parturition [26]. Day zero of pregnancy was considered the day of vaginal sperm detection. The number of positive sperm females, number of females pregnant, pregnancy index (number of pregnant females/numbers of positive sperm females), and number of cohabitation litters were reported.

### 2.5. Reproductive Organs Weights

Testes, epididymis, seminal vesicles, and prostate glands were scrutinized out and weighed from each rat. The dissected organ weight was calculated as the index weight (IW) = (organ weight (gm)/body weight (gm)) 100, as reported by Matousek [27].

### 2.6. Sperm Morphology

Sperm concentration was assayed microscopically using a hemacytometer following Yokoi et al. [28]. Cauda epididymis was diluted after mincing in 5 mL of saline, and then, the supernatant was diluted in an alkaline aqueous solution. Progressive motility, sperm, and live sperm have been evaluated (Sönmez et al. [29]). A dye exclusion approach was used to perform the viability test. For sperm abnormality evaluation, each of the epididymis contents were mixed with an eosin–nigrosin stain drop and the thin blood film scattered over slides, with a random examination of three hundred spermatozoa per slide [30].

### 2.7. Serum Testosterone Concentration Assessment

Immediately after blood was collected, sodium pentobarbital anesthesia was then left to clot. The sera were extracted at 500 RPM for 30 min by centrifugation and preserved for subsequent use at −20 °C. The blood sera utilized for testosterone measurement, according to Demetrious [31], using rats highly sensitive ELISA Kits (Immunometrics Ltd., London, UK).

### 2.8. Assays for Oxidative Stress Markers

The samples were washed three times in cold normal physiological saline solution (PBS, 0.9% NaCl). Then, the tissues were homogenized in ice-cold Tris-HCl buffer solution within a homogenizer for 2 min at 12,000× *g*. The homogenate was centrifuged at 20,000× *g* (4 °C) for 30 min, and supernatant was obtained. The levels of malondialdehyde (MDA) were tested in the homogenate. For a further extraction procedure, the supernatant was extracted in ethanol/chloroform mixture (5/3 *v*/*v*). After a second centrifugation at 3500× *g* for 20 min, the clear upper layer was taken and used for glutathione (GSH) activity determination according to Parlaktas et al. [32]. The principle of Colorimetric Evaluation was based on the response inhomogeneous form of a molecule of MDA with two thiobarbituric acid molecules resulting in a rose-colored complex with an absorbance assessed at 532 nm [33]. The GSH assay depended on reducing GSH to yield a colored complex of 5.5′-dithiobis (2-nitrobenzoic acid); its absorption was read by 405 nm within 15 min [34].

### 2.9. Testicular Pro-Inflammatory Cytokines Biomarkers

A system of quantitative sandwich enzyme immunoassay with Rat High-Sensitivity ELISA kits (Sigma-Aldrich, St. Louis, MO, USA) has been tested for testicular necrosis factor-alpha (TNF-α) and interleukin-6 (IL-6) testicular homogenates.

### 2.10. Gene Expression Analysis

The whole RNA of approximately 100 mg testicular tissue was obtained with (Invitrogen, Life Technologies, Carlsbad, CA, USA) TRIzol reagent and Nanodrop for quantification. For DNA synthesis, RNA samples of 1.8 or more A260/A280 were used using a cDNA synthesis kit (Fermentas, Waltham, MA, USA). The SYBR Green Master Mix and the primers (GAPDH) of the household gene were indicated in Table 1 added to amplify cDNA. Data on amplification were analyzed using 2^−ΔΔT^ methods [35].

### 2.11. Morphopathological Studies

Each rat’s right testis has been separated at the end of the experiment and fixed quickly in Davidson’s modified solution. Epididymis, prostate, and seminal vesicles were easily fixed for at least 24 h in 10% neutral formalin buffered. The paraffin-embedding technique was used to process the samples and to cut them into four-five µm thick. The sections were deparaffinized with xylene, stained with hematoxylin–eosin (HE), and then analyzed by light microscopy and recorded with a digital camera [36]. The testis, epididymis, prostate, and seminal vesicles damage was evaluated using a semiquantitative scoring assay, in which five random fields were examined from each section. The severity of lesions was scored and graded as follows: (−) absence of the lesion = 0%, (+) mild = 0–25%, (++) moderate = 25–50%, and (+++) severe = 50–100% of the examined tissue sections.

### 2.12. Immunohistochemistry Analysis

The Davidson-fixed rat testes use the complicated immunohistochemical process of avidin–biotin–peroxidase (Elite-ABC; Vector Laboratory, Burlingam, CA, USA) against proliferating the nuclear cell antigen (1:100 dilution; Dako Japan Co., Ltd., Tokyo, Japan) [37] was applied to positively charged paraffin tissue slides. Sections were deparaffinized, rehydrated, soaked in PBS (3–5 min), and cleaned. Then, there was a 30-min quenching of peroxidase activity utilizing 0.3% hydrogen peroxide in methyl alcohol. Samples were washed and incubated subsequently in PBS at 25 ± 1 °C and a blocking solution for ten min. Sections were incubated for 30–60 min and the primary antibody was placed in a moist chamber after rinsing with PBS (0.9% NaCl), and then it was rinsed with PBS again. Samples have been set at room temperature for 10 min with streptavidin–peroxidase and flushed with the PBS. A complex antibody–peroxidase was developed for 2–5 min using diaminobenzidine chromogen at 18–24 °C. The sections were finally cleaned up with the PBS, dehydrated, and mounted with hematoxylin by Mayer. The primary antibody had been removed or substituted by the iso-type-matched mouse IgG2a for negative controls. Positive staining microscopically is described through the visual detection of brown color. Images of 10 different fields were analyzed at a magnification of (400×) Image J. software program (ImageJ Version 1.47, National Institutes of Health, Bethesda, MD, USA for Positive Brown Immunostaining Cells estimates.

### 2.13. Data Analysis

Data have been represented as mean ± SEM. One-way variance analysis (ANOVA) was performed, which was accompanied by Duncan’s group differences detection study. The *p*-value of less than 0.05 was significantly different for all statistical analyses using “version 24” of SPSS/PC+.

## 3. Results

### 3.1. Fertility Test

The impact of FBN and BA on fertility tests are presented in Table 2. The number of positive sperm females was the lowest in FBN-administered rats. Conversely, almost all females recorded positive sperm in all other groups, and even the positive sperm females in the FBN group were not low pregnant. Moreover, the number of pregnant females and pregnancy index (%) was highest in the control and BA groups. Simultaneously, it decreased in both groups given FBN and BA (250 mg and 500 mg) and the lowest was recorded in the FBN group. The number of litters was negatively affected by FBN. Still, BA decreases this adverse effect, while the control group and BA groups (250 mg and 500 mg) were the highest in the number of litters.

### 3.2. Reproductive Organs Weights

The weight of the testes index, epididymis, and accessory sex organs in the FPN-administered group decreased dramatically. This relative index weight was retained in the group’s control values and concomitantly allocated with BA and FPN (Table 3).

### 3.3. Sperm Morphology

Sperm cell concentration, as well as motility percentage, and live sperm cell percentage were substantially (*p* ≤ 0.05) decreased, while the sperm cell abnormality percentage was considerably (*p* ≤ 0.05) improved in the FPN-administered rats concerning to the control rats (Table 4). These parameters stayed as in control, one in the groups concomitantly given with BA and FPN. Sperm abnormalities appear in the form of a bent/amorphous head and coiled/short tail. These abnormalities increased in FBN administration rats and decreased in co-administration rats.

### 3.4. Serum Testosterone, Testicular Antioxidant, and Pro-Inflammatory Cytokines

Blood serum testosterone and testicular levels of GSH in the FBN-treated group were appreciably (*p* ≤ 0.05) decreased compared with control and two levels of BA-administered rats. Concomitant administration of BA with FPN significantly increased testosterone and GSH levels but still dropped compared with the control rats for GSH. FPN-applied rats increased substantially (*p* ≤ 0.05) MDA, interleukin-6, and TNF-α concentrations compared with the control and two levels of BA groups of rats (Table 5).

### 3.5. Gene Expression

**Figure 2:**
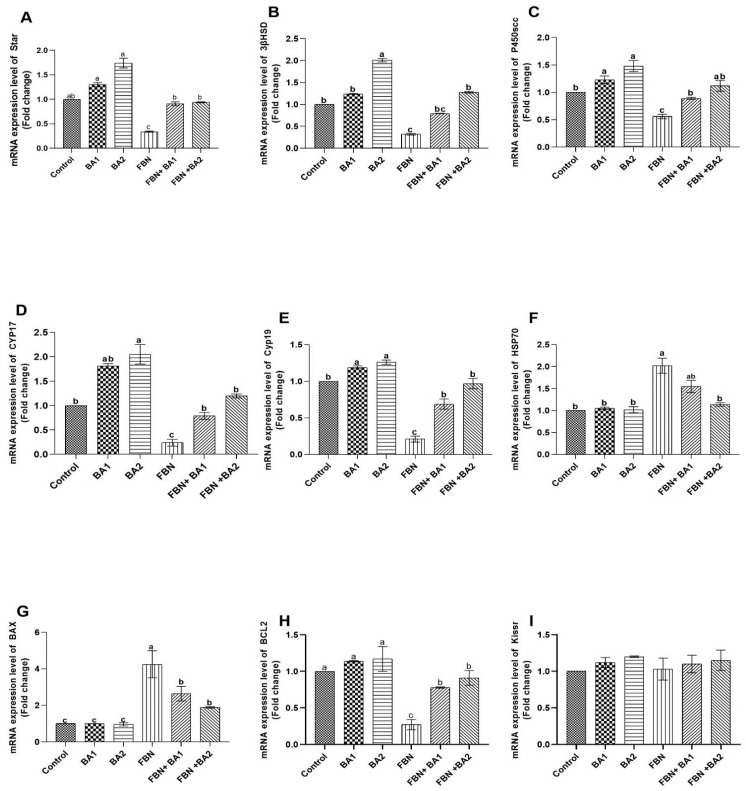
The effect of fipronil (FPN) and co-treatment with boswellic acid (BA) on gene expression of steroidogenic markers genes, apoptotic genes, and heat shock protein gene. STAR (**A**), 3β-HSD (**B**), P450SCC (**C**), CYP17A1 (**D**), Cyp19 (**E**), HSP70 (**F**), Bax (**G**), Bcl-2 (**H**), and KISS1 (**I**). All values are expressed as mean ± SEM. (**a**–**c**) Mean values of different letters within the same row are significantly different (*p* < 0.05, ANOVA with Duncan’s multiple range test).

FPN-administered rats showed significant downregulation in steroidogenic and fertility-related gene expression, including *CYP17A1* and cytochrome *P450 17A1 KISS1*, *kisspeptin*, *STAR*, *Cyp11a1*, cholesterol side-chain cleavage enzyme mRNA (*P450SCC*) *Hsd3b1*, 3-beta-hydroxysteroid dehydrogenase/delta-5-delta-4 isomerase type I, *Cyp19*, and cytochrome P450 aromatase, concerning other administered groups and the control one as shown in Figure 2. Interestingly, co-administration of BA with FPN showed significant upregulation and stabilization of the steroidogenic gene expression, as presented in Figure 2. In addition, the FPN-administered groups showed a considerable increase in Bax, gene expression, and heat shock protein with significant downregulation to Bcl-2 gene expression concerning other administered groups in which the BA groups showed significant normalization of these genes concerning the control group. The kisspeptin gene showed no substantial changes between all treated groups.

### 3.6. Histopathological Findings

There were no histological architecture differences among the control and two levels of BA (250–500 mg/kg) groups in the examined tissues. The incidence and severity of histopathological findings in the examined testis, epididymis, prostate gland, and seminal vesicle of Fipronil and co-treatment with boswellic acid are summarized in Table 6.

### 3.7. Testicular Tissue

**Figure 3:**
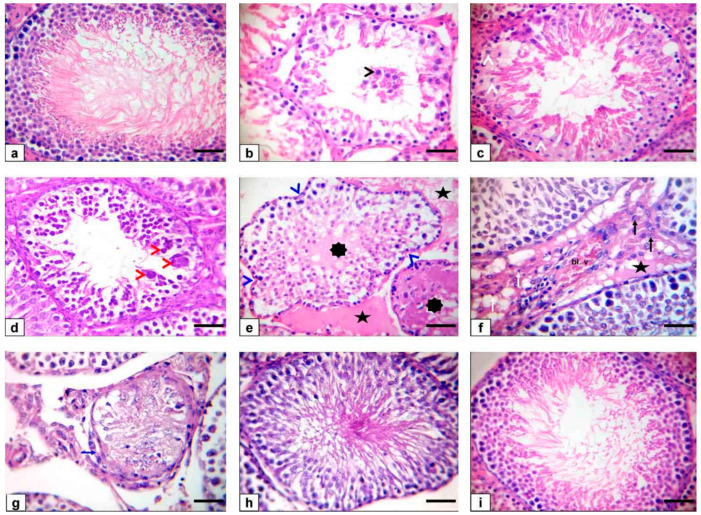
Photomicrograph of rat testes stained with HE. Bar = 50 μm. (**a**) Normal testes histoarchitecture of control rats (**b**–**g**) FPN-administered rats showing sloughing of the germinal epithelium in the lumen of seminiferous tubules (black arrowhead), fragmentation or necrosis of tubular epithelium (white arrowheads) with giant cell formations in the lumen of seminiferous tubules (red arrowheads), depletion of germinal cells and hyalinization of the luminal contents (asterisks) besides shrunken, buckled, and disorganized seminiferous tubules (blue arrowheads) as well as interstitial edema (stars), mild inflammatory cell infiltration (white arrows), mild hyperplasia of interstitial endocrine cells (black arrows) with congestion of the interstitial blood vessels (Bl. v), and finally atrophy of some seminiferous tubules (blue arrow). (**h**) FPN + BA_1_ rats showing the normal histoarchitecture of seminiferous tubules with the presence of spermatids and spermatozoa in their lumen. (**i**) FPN + BA_2_ rats showing normal histoarchitecture of seminiferous tubules.

The control rats’ testicular tissue showed regular, uniform, well-organized seminiferous tubules and normal interstitial connective tissue with entire spermatogenesis (Figure 3a). Testes of the FPN-administered group exhibited exfoliation of the germinal epithelium in the lumen of seminiferous tubules (Figure 3b) as well as necrosis of tubular epithelium (Figure 3c) with multinucleated giant cell formations in some seminiferous tubule’s lumen (Figure 3d). Moreover, some tubules showed reduced germinal cells and coagulative necrosis with luminal content hyalinization, in addition to the degenerative changes of most of the seminiferous tubules as diminished or collapsed. Incoherent seminiferous tubules (Figure 3e) were noticed. Furthermore, interstitial connective tissue exhibited edema defined by faint eosinophilic substances, mild inflammatory cell infiltration, and mild interstitial endocrine cell hyperplasia with interstitial vessel obstruction (Figure 3f). Finally, it atrophied some seminiferous tubules (Figure 3g), which was characterized by tiny seminiferous tubules, and thickened basement membrane and marked reduced numbers of germinal cells. Conversely, rats that received FPN plus BA (250 and 500 mg/kg) showed major enhancement of most seminiferous tubules’ spermatogenesis by the inclusion of elongated spermatids and spermatozoa (Figure 3h,i).

### 3.8. Epididymis

**Figure 4:**
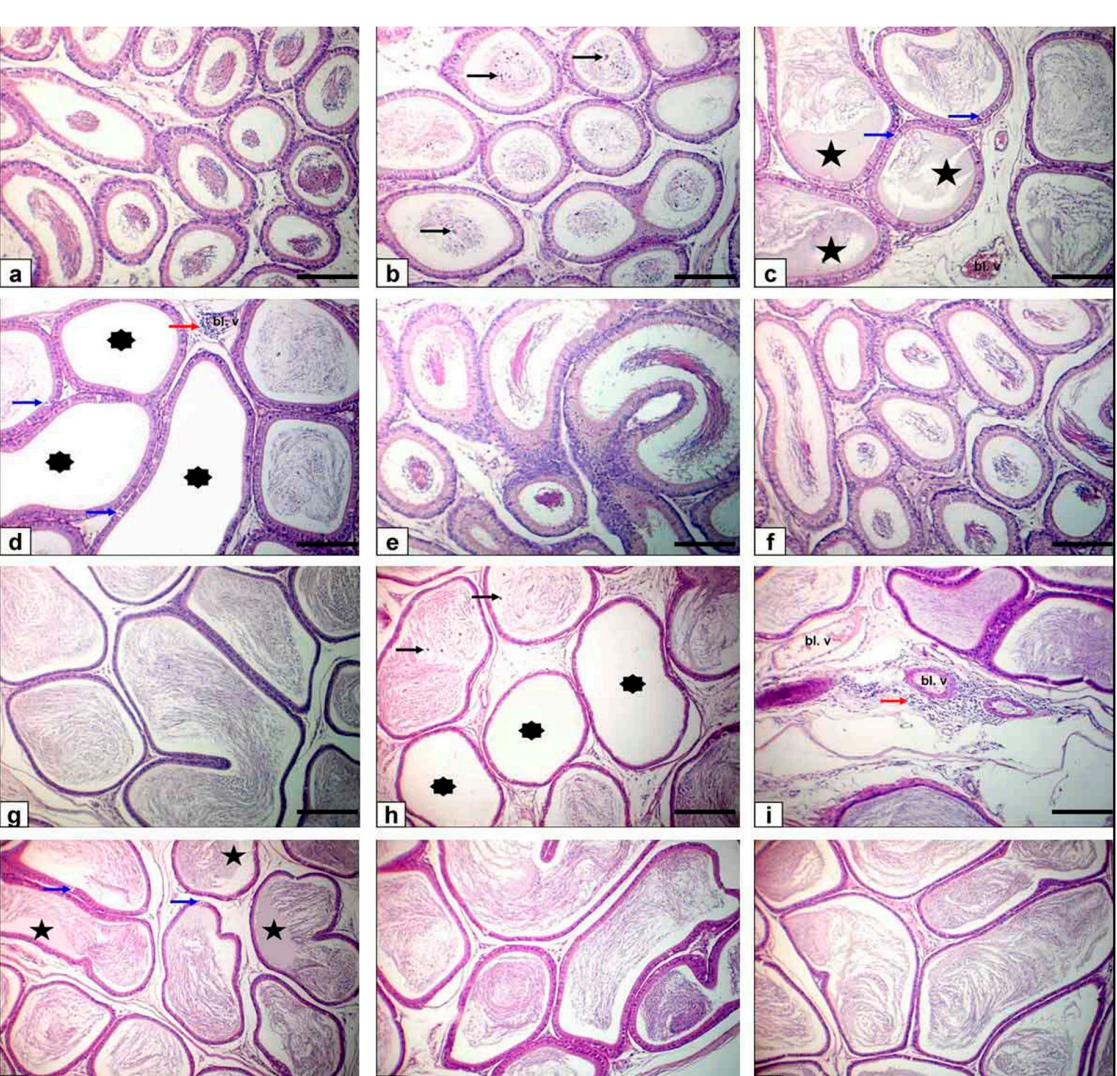
Photomicrograph of rat epididymis stained with HE. Bar = 100 μm. (**a**–**f**) Caput epididymis (**g**–**l**) cauda epididymis. (**a**,**g**) Normal histological structure with normal sperm density of control rats. (**b**–**d**,**h**–**j**) FPN-treated rats showing sloughing of some germinal epithelial cells (black arrows), vacuolation of few germinal epithelial cells (blue arrows), interstitial congestion of blood vessel (Bl. v) with interstitial and perivascular inflammatory cell infiltrations (red arrow) in addition to low or/and free (asterisks) sperm density with hyalinization of the luminal contents (stars) of some ductal epididymis. (**e**,**k**) FPN + BA_1_ rats showing normal structure with the marked enhancement of sperm density. (**f**,**l**) FPN + BA_2_ rats showing normal structural integrity and sperm density.

In the control group, the caput and cauda epididymis exhibited ordinary sperm intensity histological architecture. The caput and cauda epididymis of FPN-administered rats showed histopathological alterations as a shedding of germinal cells in their lumen, vacuolation of few germinal epithelial cells, interstitial blood vessels congestion with interstitial and perivascular inflammatory cell permeations beside low or/and free sperm density, and luminal content hyalinization of some ductal epididymis. Epididymal ductules of FPN plus BA showed typical histological architecture with marked enhancement in sperm density (Figure 4).

### 3.9. Prostate Gland

**Figure 5:**
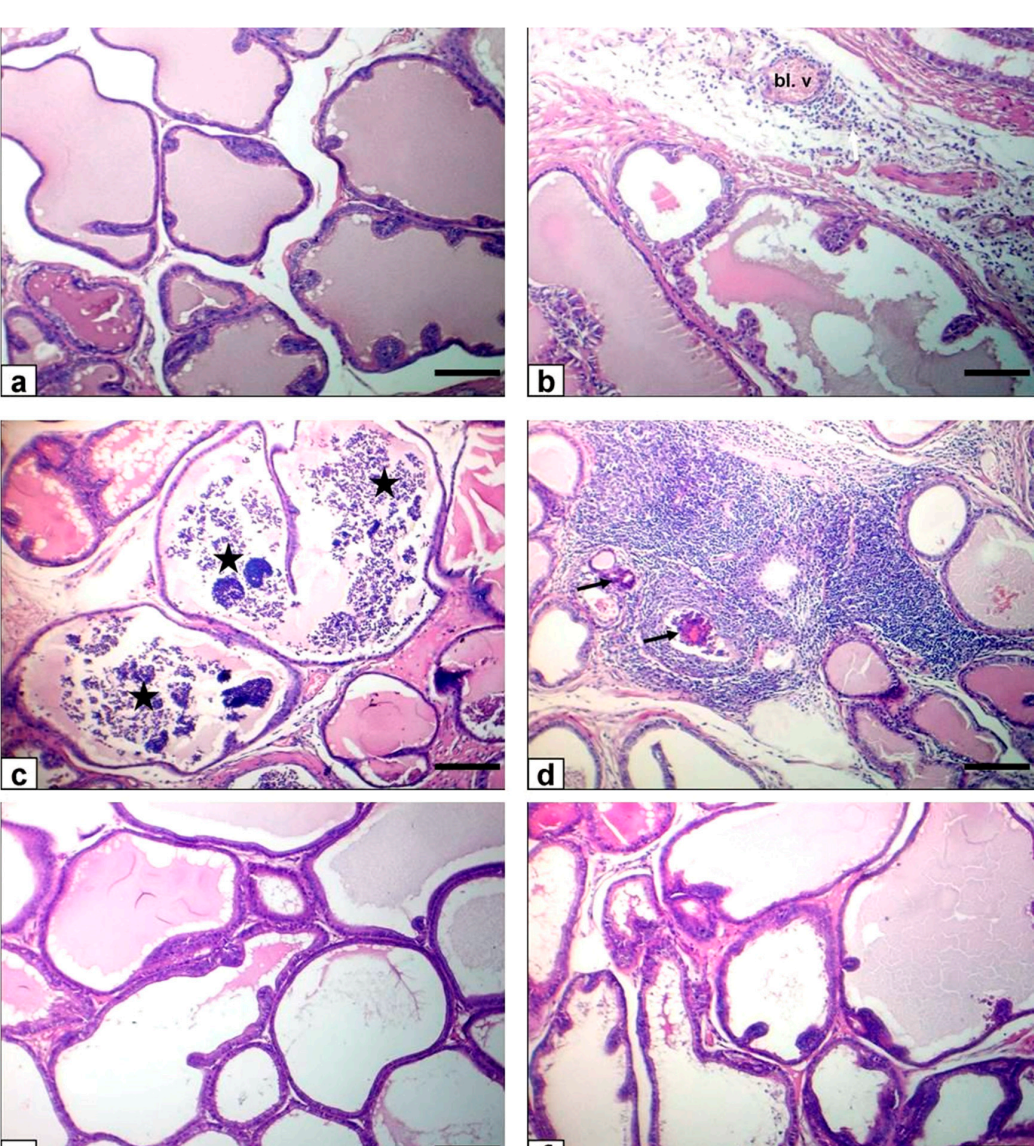
Photomicrograph of rat prostate stained with HE. Bar = 100 μm). (**a**) A prostate of control rats with the normal histological structure of the glandular epithelium and normal luminal secretions. (**b**–**d**) FPN-treated rats showing interstitial congestion (Bl. v) and perivascular inflammatory cell infiltration (white arrow) with low luminal secretions beside the desquamation of glandular epithelium (stars) and severe leukocytes in the interstitium with necrosis of glandular acini (black arrows). (**e**) FPN + BA_1_ rats showing normal histoarchitecture of glandular epithelium and moderate luminal secretions. (**f**) FPN + BA_2_ rats showing nearly normal histoarchitecture and moderate luminal secretions.

The control group’s prostate revealed the typical histological arrangement of the glandular epithelium and normal luminal secretions (Figure 5a). FPN-administered rats showed interstitial congestion and perivascular inflammatory cell infiltration with low luminal secretions (Figure 5b) in addition to glandular epithelial desquamation (Figure 5c) and severe white blood cells infiltration with necrosis of glandular acini (Figure 5d). The prostate gland of FPN plus BA showed normal histoarchitecture of glandular epithelium and moderate luminal secretions (Figure 5e,f).

### 3.10. Seminal Vesicle

**Figure 6:**
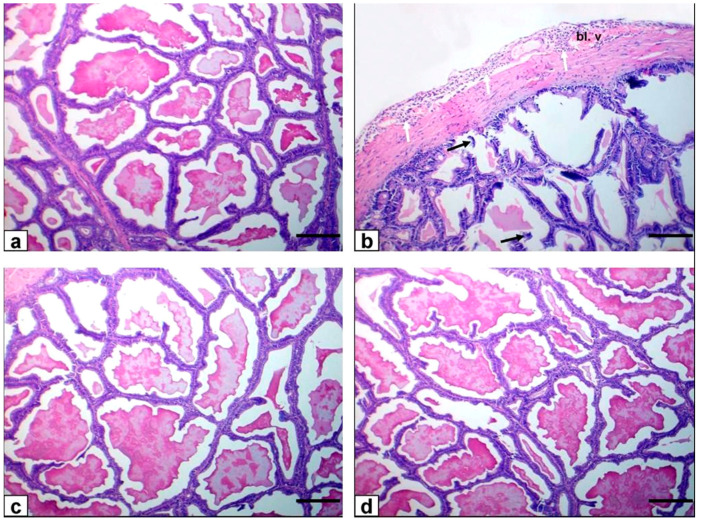
Photomicrograph of rat seminal vesicle stained with HE. Bar = 100 μm). (**a**) The seminal vesicle of the control rats with normal structure and normal luminal secretions. (**b**) FPN-treated rats showing neutrophils, plasma cells, and lymphocytes infiltration in the tunica serosa and the tunica muscularis (white arrows) beside the congestion of blood vessel (Bl. v) and desquamation of some necrotic tubuloalveolar glandular epithelial cells (black arrows) with low luminal secretions. (**c**) FPN + BA_1_ rats showing nearly normal histoarchitecture. (**d**) FPN + BA_2_ rats showing nearly normal histoarchitecture.

Our study showed that the control rats’ seminal vesicle had a standard structure and normal luminal secretions (Figure 6a). FPN-administered rats exhibited seminal vesiculitis expressed in the serosa tunic and muscular tunica by white blood cell infiltrations, mainly neutrophils, plasma cells, and lymphocytes, besides congestion of blood vessels desquamation of specific necrotic epithelial glandular cells with low luminal secretions (Figure 6b). The seminal vesicle of FPN plus BA showed nearly normal histoarchitecture (Figure 6c,d).

### 3.11. Immunohistochemistry and Quantitative Analysis

**Figure 7:**
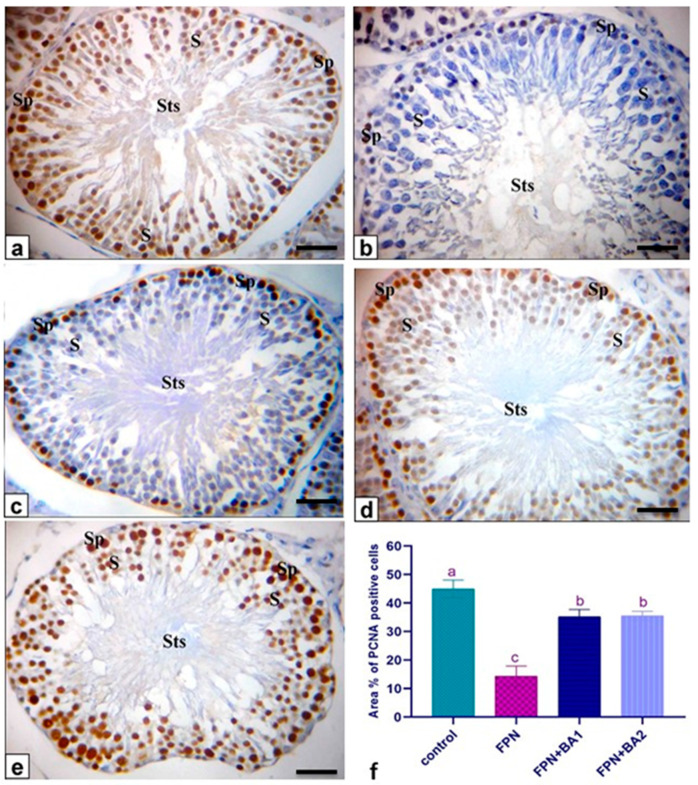
Photomicrograph of rat seminiferous tubules (Sts) showing positive brown proliferating cell nuclear antigen (PCNA) immunostaining. Bar = 50 μm. (**a**) Control rat showing positive brown PCNA immunoreactions in the nuclei of spermatogonia (Sp) and spermatocytes (S). (**b**,**c**) FPN-treated rats showing that most of the seminiferous tubules have negative immunostained in the nuclei of spermatogonia and spermatocytes. In contrast, other seminiferous tubules have few PCNA immunoreactions in the nuclei of the spermatogenic cells and negative spermatocytes. (**d**) FPN + BA_1_ rats were showing positive brown PCNA immunoreactions in the nuclei of spermatogonia and spermatocytes (**b**) FPN + BA_2_ rats showing positive brown PCNA immunoreactions in the nuclei of spermatogonia and spermatocytes. (**f**) Area percentage of PCNA positive brown-stained cells. All the values were expressed as mean ± SEM. Different small letters indicate significant at *p* < 0.0001. (**a**–**c**) Mean values with different letters within columns are significantly different at *p* ≤ 0.05 (ANOVA) with Duncan’s multiple range test.

The proliferating cell nuclear antigen (PCNA) is useful for assessing germ-cell kinetics, especially for pathological diagnosis of germinal arrest, difficult to differentiate by the HE staining technique. The control rat’s seminiferous tubules exhibited positive brown nuclei of spermatogonia and spermatocytes PCNA immunoreactions (Figure 7a). FPN-administered rats showed that most seminiferous tubules have negative immune-stained spermatogonia and spermatocyte nuclei (Figure 7b). In comparison, other seminiferous tubules have few positive brown PCNA nuclei immunoreactions of the spermatogenic cells and negative spermatocytes (Figure 7c). The seminiferous tubules of FPN plus BA showed positive brown PCNA immunoreactions in the spermatogonia and spermatocytes nuclei (Figure 7d,e). The quantitative analysis indicated a marked decline in the area percentage of PCNA immunopositive cells in FPN-administered rats concerning the control one. Co-administration with BA showed a decrease in the area percentage of PCNA immunopositive cells (Figure 7f).

## 4. Discussion

Chemical insecticides are widely utilized worldwide in the agriculture sector and for other purposes [38]. Food residues, contaminated tap water, occupational exposure, repellence, household use, and application against fleas and ticks are various sources for endangering insecticides for animals and people [39,40]. FPN is an insecticide with phenylpyrazole in chemical form. It is a common insecticide used both in agriculture and in domestic pest management [25]. However, few studies are evaluating its consequence on the fertility of males and reproductive efficiency. Therefore, this experiment was conducted to determine the effects of FPN on fertility test, the weight of the male reproductive organ, seminal study, serum testosterone level, oxidative status, cytokines level, some gene expression, and histopathology and to evaluate the ameliorative effect of BA. In our study, FPN has a distinct adverse effect on fertility tests by decreasing pregnant females, pregnancy index, and many litters.

Moreover, it gave rise to a noteworthy decrease in the reproductive organs’ weights due to the decline in serum testosterone concentration, sperm quantity, sperm progressive motility, and live sperm cell percentage. It considerably boosted sperm cell abnormalities percentage in the FPN-administered group, which many attribute to its hazard impact. Consequently, the findings obtained indicate that FPN decreases sperm cell quality, leading to male rats’ infertility. The pathway of insecticide toxicity on the testicular tissue can be correlated with the activation of oxidative injuries. These results agree with Mazzo, Balieira, Bizerra, and Mingatto [6], who reported that FPN-induced harmful impacts on sperm quality. Sperm motility is impaired by FPN’s long-term exposure [11]. In addition, the overproduction of reactive oxygen species (ROS) exceeds the cellular capability, leads to oxidative damage, and reduces sperm viability and fertility [1]. The sperm contains a large proportion of polyunsaturated fatty acids and is highly susceptible to harm caused by excessive oxidative damage and peroxidation to its plasma membrane, leading to a loss of motility and decreased number [41,42]. FPN-administered rats showed a remarkable increase in MDA, which considers the consequence of lipid peroxidation and lipid degradation triggering radicals and reduction of GSH levels, which reverberate the degree of oxidative harm. These results are following the work of Mossa et al. [43] that demonstrated decreased concentrations of GSH in the kidneys and liver of rats handled with FPN (10 mg/kg bwt) [44]. The reduction in the content of GSH in the kidney and brain of FPN-treated mice (5 and 10 mg/kg bwt) in the same FPN-treated line (5 mg/kg bwt) resulted in a decrease in the concentration of GSH in the testis [6]. These results are probably attributed to Fipronil’s oxidative anxiety, including reduced GSH levels, and antioxidant activity and consequent lipoperoxidation [45]. FPN-administered rats showed an overexpression of TNF-α and IL-6 pro-inflammatory cytokines. TNF-α is a major inflammatory and immune response cytokine [46], and IL-6 is a cytokine pleiotropic rendered by macrophages of tissue and monocytes [47].

The utilized doses of boswellic acid in the current study were tested previously as reported by Sami et al. [21] in which they found that boswellic acids can ameliorate doxorubicin-induced nephrotoxicity in mice, they used different doses of boswellic acid (125 mg/kg), (250 mg/kg), and (500 mg/kg), and they found that the effect of the high dose of BAs (500 mg/kg) was different (more ameliorative) from that observed with the lowest dose (125 mg/kg). In the same line, Nusier et al. [20] studied the effect of two different doses of boswellic acid: 250 and 500 mg/kg. They reported variation in their impact on the reproductive system of the rat. Barakat et al. [22,23] investigated the protective effect of boswellic acid in different doses 250 and 500 in doxorubicin-induced hepatic damaged. The anti-aggregatory effect of boswellic acid in high-fat fed rats found a variation in the boswellic acid in a dose-dependant manner. Al-Yahya et al. [24] reported that the boswellic acid is safe up to 1000 mg/kg in rats. Still, this dose is relatively high considering the amount of extract consumed by humans. So, in this study, we try to investigate the ameliorative effect of boswellic acid on the toxic impact of fipronil in a dose–response manner.

The impact of FBN and BA on fertility tests is presented in Table 2. Almost all females recorded positive sperm in all other groups other than the FBN group; moreover, the pregnancy index (%) was highest in the control and BA groups. Still, BA decreases this adverse effect, while the control group and BA groups (250 mg and 500 mg) were the highest in the number of litters. The obtained results were inconsistent with [20]. They reported that oral administration of boswellic acid increased the fertility in rats and the number of implantations as well as increased spermatogenesis due to its antioxidant activity. In the same line, our work was in harmony with [20], in which they reported non-significant improvement in the fertility parameters in BA (500 mg/kg) dose concerning (250 mg/kg). The concomitant administration of both doses of BA with FPN significantly increases testosterone and GSH with a significant decrease in MDA, interleukin-6, and TNF-α concentrations concerning the FBN-treated group, as shown in Table 5. with non-significant improvement in the BA higher dose concerning the lower dose of BA. The obtained result was in the same line with Sami et al. [21] in which they reported the nephroprotective effect of BA with a non-significant improvement of the higher dose of BA (500 mg/kg) due to the antioxidant and antiapoptotic effect of boswellic acid.

The results showed that FPN-administered rats showed a significant downregulation in steroidogenic and fertility-related gene expression, including *CYP17A1* and *cytochrome P450 17A1*, *KISS1*, *kisspeptin*, *STAR*, *Cyp11a1*, cholesterol side-chain cleavage enzyme mRNA (*P450SCC*) *Hsd3b1*, 3-beta-hydroxysteroid dehydrogenase/delta-5-delta-4 isomerase type I, *Cyp19*, and cytochrome P450 aromatase compared with other administered groups and the control one. However, co-administration of BA (both doses) with FPN showed significant upregulation and stabilization of the steroidogenic gene expression. Steroid hormones are produced from cholesterol through various steroid cytochrome P450 hydroxylases-induced reactions [48,49]. Cholesterol transfer from the external to the mitochondria’s inner membrane by steroidogenic acute regulating protein (StAR) involves a rate-restricted steroidogenesis phase [50]. Then, steroidogenesis begins with the transition of P450 (P450scc/CYP11A1/Cyp11a1) cholesterol to pregnenolone, which is an essential molecule in developing the body steroid hormones [48].

Light microscope examination of FPN-administered rats testes showed degenerated and exfoliated germ cells in the seminiferous tubular lumen, which was attributed by the main effect of the cell-to-cell junction of Sertoli and germ cells or microtubular deterioration that lead to Sertoli cell damage [51,52]. In the current work, fragmentation or necrosis of tubular epithelium with giant cell formations in the seminiferous tubular lumen, germ cell loss, and luminous hyalinization dwindled, collapsed, and incoherent seminiferous tubules were reported. FPN caused interstitial edema because of increased vascular permeability and mononuclear cell infiltration that secrete cytokines, causing congested interstitial blood vessels. Another important finding in atrophied seminiferous tubules was noticed in our study due to the cytotoxic effect of FPN. The epididymal lesions were sloughing off some germinal epithelial in the lumen of some ducts of the epididymis, which indicate testicular malfunction [53,54] and interstitial congestion of blood vessel with perivascular inflammatory cell infiltrations; besides, most epididymal ducts seemed to have no or low sperm quantities in their lumen, which reflected the cessation of spermatogenesis. The prostates and seminal vesicle lesions experienced necrosis and desquamation of some glandular epithelial cells with low luminal discharges and severe interstitial leukocytes infiltration [53]. These alterations may be due to reduced testosterone, which needs differentiation, development, and the preservation of epithelial cells of accessory sex glands [55]. To estimate germ cell kinetics and an indication of DNA synthesis deterioration, PCNA is considered a valuable molecular marker [56]. Similarly, it measures the cell proliferation and spermatogenic role of studies in male infertility. In spermatogonia and the proliferating control rat spermatocytes, many positive brown nuclear reactions have been found, while a reduced countenance and area percentage of PCNA antibody was detected in FPN-administered rats compared with the control group. There were no histological architecture differences among the control and two levels of BA (250–500 mg/kg) groups in the examined tissues. Conversely, rats that received FPN plus BA (250 and 500 mg/kg) showed significant enhancement of most seminiferous tubules’ spermatogenesis by including elongated spermatids and spermatozoa. These results were inconsistent with [21] in which they reported that the most convenient restoration of these parameters was achieved in the doxorubicin + BA 500 mg/kg group. Supporting our result, data obtained by Kruger et al. [57] have reported antioxidants and chelate metals of BAs in oxidative injury pathways.

The co-administration of BA significantly improved the evaluated parameters such as the number of pregnant rats, pregnancy index, and the number of litters, reproductive organs weights, normalized testosterone levels, and sperm quality, which were attributed to the increasing number of spermatocytes and normal spermatogenesis as found by Nusier et al. [20], which reported the promising effect of boswellia on adult male rat fertility due to its antioxidant activity [19]. BA treatment (250 and 500 mg/kg) normalizes the apoptotic effect of FPN, which is cleared by our result, in which FPN significantly upregulates Bax, heat shock protein, and gene expression with significant downregulation to Bcl-2. These results show inconsistency with [21]; they revealed the antioxidant and antiapoptotic effects of BA. BA’s prospective role in the scavenging of FPN ROS was linked in histological architecture to testes, the epididymis, and sexual accessory glands. Upon our result, the molecular mechanism of FBN that induced infertility was summarized in the downregulation in steroidogenic and fertility-related gene expression, including *CYP17A1* and *cytochrome P450 17A1*, *KISS1*, *kisspeptin*, *STAR*, *Cyp11a1*, cholesterol side-chain cleavage enzyme mRNA (*P450SCC*) *Hsd3b1*, 3-beta-hydroxysteroid dehydrogenase/delta-5-delta-4 isomerase type I, *Cyp19*, and cytochrome P450 aromatase. In addition, FPN-treated rats showing few PCNA immunoreactions in the nuclei of the spermatogenic cells and negative spermatocytes FPN caused interstitial edema because of increased vascular permeability and mononuclear cell infiltration that secrete cytokines *IL-6* and *TNF-α*, causing congested interstitial blood vessels. Another important finding regarding atrophied seminiferous tubules was noticed in our study due to the cytotoxic effect of FPN blood serum testosterone, and testicular levels of GSH in FBN-treated group were appreciably (*p* ≤ 0.05) decreased.

Many studies are concerned with the significant mechanism driving the protective actions in which boswellic acid-treated rats showed reduced serum expression of *TNF-α* and *IL-6* and hepatic iNOS. At the cellular level, also, boswellic acids increased the expression in the white adipose tissue of thermogenesis associated mitochondrial uncoupling protein-1 and carnitine palmitoyl transferase-1 [58].

Gayathri et al. [59] showed that boswellic acid has anti-inflammatory effects in human peripheral mononuclear blood cells and mouse macrophages by inhibiting *tumor necrosis factor-α* (*TNF-α*), *IL-1β*, *nitric oxide*, and mitogenic protein kinases. Several clinical trials highlighted boswellic acid as a potentially effective anti-inflammatory drug [60]. In addition, BA extract inhibited the *TGF-ß*-induced fibrosis (*p* = 0.01) and 5-lipooxygenase activity levels that prevent fibrosis, as mentioned by Ali and Mansour [61]. In the same line, Sharma, et al. [62] reported the anti-inflammatory role of the boswellic acid through the inflammatory mediators *TNF-α* and *IL-6*. The inhibition of *NF-κB* activity by the boswellic acid family may be considered an alternative therapy for chronic inflammatory disorders [63].

## 5. Conclusions

BA significantly improved the reproductive parameters assessed, such as the number of pregnant females, index of pregnancy and the number of litters, weights of the reproductive organ, sperm cell quality, morphological alterations of testes, epididymis, and sex glands by accessory caused by FPN oxidative stress, as well as the improvement of steroidogenesis, antioxidants, and antiapoptotic marker. This is the first report to address the defensive function of BA in male rats against reproductive FPN lesions.

Fipronil (FPN) is a commonly used phenylpyrazole pesticide used to manage insects and remove fleas, ticks, and other parasites. While FPN poses health risks, it is frequently encountered in everyday life. Taken together, FPN can restrain various sperm functions directly and indirectly. Thus, FPN can adversely affect male fertility, which leads to infertility. We suggest that using FPN as a pesticide demands the attention of reproductive toxicity from these findings.

## Figures and Tables

**Table 1:** Primers for gene expression by RT-PCR.

Gene	Direction	Primer Sequence	Accession Number
*Bax*	Sense	GGCGAATTGGCGATGAACTG	NM_017059.2
	Antisense	ATGGTTCTGATCAGCTCGGG	
*Bcl-2*	Sense	GATTGTGGCCTTCTTTGAGT	NM_016993.1
	Antisense	ATAGTTCCACAAAGGCATCC	
*HSP70*	Sense	TCAGAGCTGCTATGTCGCTG	NM_153629.1
	Antisense	GCAGCGGTCGCTATACTCAT	
*CYP17A1*	Sense	ACTGAGGGTATCGTGGATGC	NM_012753.2
	Antisense	TCGAACTTCTCCCTGCACTT	
*StAR*	Sense	CTGCTAGACCAGCCCATGGAC	NM_031558.3
	Antisense	TGATTTCCTTGACATTTGGGTTCC	
*KISS1*	Sense	TGCTGCTTCTCCTCTGTGTGG	NM_181692.1
	Antisense	ATTAACGAGTTCCTGGGGTCC	
*Cyp11a1*	Sense	AGGTGTAGCTCAGGACTT	J05156
	Antisense	AGGAGGCTATAAAGGACACC	
*3β-HSD*	Sense	CCCATACAGCAAAAGGATGG	M38178
	Antisense	GCCGCAAGTATCATGACAGA	
*Cyp19*	Sense	GCTTCTCATCGCAGAGTATCCGG	M33986
	Antisense	CAAGGGTAAATTCATTGGGCTTGG	
*GAPDH*	Sense	TCAAGAAGGTGGTGAAGCAG	NM_017008.4
	Antisense	AGGTGGAAGAATGGGAGTTG	

*Bax*, Bcl-2-associated X protein. *Bcl-2*, B-cell lymphoma 2. *CYP17A1*, cytochrome P450 17A1. *GAPDH*, glyceraldehyde-3-phosphate dehydrogenase. *HSP70*, heat shock protein 70. *KISS1*, kisspeptin. *StAR*, steroidogenic acute regulatory protein. *Cyp11a1*, cholesterol side-chain cleavage enzyme mRNA (P450SCC). *3β-HSD*, 3-beta-hydroxysteroid dehydrogenase/delta-5-delta-4 isomerase type I; *Cyp19*, cytochrome P450 aromatase.

**Table 2:** The effect of fipronil (FPN) and co-treatment with boswellic acid (BA) on fertility index of rat.

Group	Control	BA_1_	BA_2_	FBN	FBN + BA_1_	FBN + BA_2_
No. of females	12	12	12	12	12	12
No. of positive sperm female	10/12 (83.3%)	11/12 (91.7%)	11/12 (91.7%)	4/12 (33.3%)	9/12 (75%)	10/12 (83.3%)
No. of pregnant female	9/12 (75%)	10/12 (83.3%)	11/12 (91.7%)	3/12 (25%)	7/12 (58.3%)	8/12 (66.7%)
Pregnancy index (%)	90	90.9	100	75	77.8	80
No. of litters	8.52 ± 1.43 ^c^	10.36 ± 2.01 ^b^	12.86 ± 2.85 ^a^	3.58 ± 0.98 ^e^	5.63 ± 1. 56 ^de^	6.09 ± 1.74 ^d^

The data shown are the mean and standard deviation. ^a–e^ Means bearing different superscript letters within the same row are significantly different (*p* < 0.05). Group 1 (control); Group 2 (BA1) rats were orally administered 250 mg BA/kg bwt; Group 3 (BA2) rats were orally administered 500 mg BA/kg bwt; Group 4 (FPN) rats were orally administered 20 mg FPN/kg bwt; Group 5 (FPN + BA1) rats were orally administered 20 mg FPN/kg bwt plus 250 mg BA/kg bwt, and Group 6 (FPN + BA2) rats were orally administered 20 mg FPN/kg bwt plus 500 mg BA/kg bwt.

**Table 3:** The effect of fipronil (FPN) and co-treatment with boswellic acid (BA) on the index weight of rat reproductive organs.

Groups/Parameters	Control	BA_1_	BA_2_	FBN	FBN + BA_1_	FBN + BA_2_
I.W. of testes	1.67 ± 0.05 ^a^	1.63 ± 0.04 ^a^	1.64 ± 0.06 ^a^	1.17 ± 0.04 ^b^	1.57 ± 0.07 ^a^	1.58 ± 0.07 ^a^
I.W. of epididymis	0.82 ± 0.01 ^a^	0.80 ± 0.02 ^a^	0.83 ± 0.02 ^a^	0.65 ± 0.02 ^b^	0.79 ± 0.01 ^a^	0.78 ± 0.02 ^a^
I.W. of accessory gland	0.94 ± 0.03 ^a^	0.95 ± 0.04 ^a^	0.96 ± 0.02 ^a^	0.77 ± 0.02 ^b^	0.90 ± 0.02 ^a^	0.91 ± 0.03 ^a^

All values are expressed as mean ± S.E. ^a–b^ Mean values with different letters at the same raw are significantly different at *p* ≤ 0.05 (ANOVA) with Duncan’s multiple range test. Group 1 (control); Group 2 (BA1) rats were orally administered 250 mg BA/kg bwt; Group 3 (BA2) rats were orally administered 500 mg BA/kg bwt; Group 4 (FPN) rats were orally administered 20 mg FPN/kg bwt; Group 5 (FPN + BA1) rats were orally administered 20 mg FPN/kg bwt plus 250 mg BA/kg bwt, and Group 6 (FPN + BA2) rats were orally administered 20 mg FPN/kg bwt plus 500 mg BA/kg bwt.

**Table 4:** The effect of fipronil (FPN) and co-treatment with boswellic acid (BA) on semen analysis.

Groups/Parameters		Control	BA_1_	BA_2_	FBN	FBN + BA_1_	FBN + BA_2_
Sperm cell count (×10^6^/mL)		150.40 ± 1.51 ^a^	149.50 ± 2.42 ^a^	152.50 ± 1.50 ^a^	112.20 ± 1.49 ^b^	145.00 ± 1.71 ^a^	146.40 ± 1.51 ^a^
Sperm motility %		90.00 ± 1.22 ^a^	90.00 ± 3.74 ^a^	91.00 ± 3.67 ^a^	74.00 ± 2.92 ^b^	88.00 ± 1.87 ^a^	89.00 ± 1.87 ^a^
Live spermatozoa %		92.00 ± 2.22 ^a^	91.00 ± 1.74 ^a^	90.00 ± 2.67 ^a^	82.00 ± 1.92 ^b^	90.00 ± 2.02 ^a^	91.00 ± 1.92 ^a^
Abnormality %		8.20 ± 0.51 ^b^	8.00 ± 0.58 ^b^	8.33 ± 0.33 ^b^	14.30 ± 0.71 ^a^	8.50 ± 1.00 ^b^	8.63 ± 0.88 ^b^
Abnormalities							
1	Bent head	1.60 ± 0.24 ^b^	1.67 ± 0.33 ^b^	2.00 ± 0.58 ^a^	2.80 ± 0.65 ^a^	2.33 ± 0.33 ^a^	2.17 ± 0.33 ^a^
2	Amorphous head	1.80 ± 0.51 ^b^	2.00 ± 0.58 ^b^	1.33 ± 0.33 ^d^	4.00 ± 0.71 ^a^	1.67 ± 0.33 ^c^	2.00 ± 0.03 ^b^
3	Coiled tail	1.80 ± 0.58 ^c^	2.33 ± 0.33 ^b^	2.38 ± 0.88 ^b^	3.25 ± 0.25 ^a^	2.17 ± 0.88 ^b^	2.35 ± 0.88 ^b^
4	Short tail	3.00 ± 0.55 ^b^	2.00 ± 0.58 ^c^	2.67 ± 0.33 ^c^	4.25 ± 0.85 ^a^	2.33 ± 0.88 ^c^	2.13 ± 0.33 ^c^

All values are expressed as mean ± S.E. ^a–d^ Mean values with different letters at the same raw are significantly different at *p* ≤ 0.05 (ANOVA) with Duncan’s multiple range test. Group 1 (control); Group 2 (BA1) rats were orally administered 250 mg BA/kg bwt; Group 3 (BA2) rats were orally administered 500 mg BA/kg bwt; Group 4 (FPN) rats were orally administered 20 mg FPN/kg bwt; Group 5 (FPN + BA1) rats were orally administered 20 mg FPN/kg bwt plus 250 mg BA/kg bwt, and Group 6 (FPN + BA2) rats were orally administered 20 mg FPN/kg bwt plus 500 mg BA/kg bwt.

**Table 5:** The effect of fipronil (FPN) and co-treatment with boswellic acid (BA) on serum testosterone, antioxidant, and pro-inflammatory cytokines.

Groups/Parameters	Control	BA_1_	BA_2_	FBN	FBN + BA_1_	FBN + BA_2_
Testosterone (ng/mL)	2.43 ± 0.042 ^a^	2.31 ± 0.022 ^a^	2.29 ± 0.039^a^	1.63 ± 0.023 ^b^	2.34 ± 0.032 ^a^	2.22 ± 0.025 ^a^
MDA (nmol/mg protein)	47.50 ± 0.76 ^c^	48.00 ± 0.58 ^c^	48.17 ± 0.60 ^c^	71.83 ± 0.95 ^a^	55.83 ± 0.60 ^b^	56.50 ± 0.76 ^b^
GSH (mmol/mg protein)	42.33 ± 0.67 ^a^	43.50 ± 0.76 ^a^	43.83 ± 0.60 ^a^	19.17 ± 0.60 ^d^	30.67 ± 0.49 ^c^	33.17 ± 0.60 ^b^
IL-6 (pg/mL)	102.00 ± 0.97 ^c^	103.50 ± 1.15 ^c^	103.33 ± 0.88 ^c^	203.00 ± 0.97 ^a^	138.63 ± 1.70 ^b^	139.17 ± 0.60 ^b^
TNF-α (pg/mL)	76.80 ± 0. 60 ^c^	77.50 ± 0.76 ^c^	78.50 ± 0.60 ^c^	151.33 ± 1.05 ^a^	99.50 ± 0.76 ^b^	100.50 ± 0.76 ^b^

All values are expressed as mean ± SEM. ^a–c^ Mean values of different letters within the same row are significantly different (*p* < 0.05, ANOVA with Duncan’s multiple range test). Group 1 (control); Group 2 (BA1) rats were orally administered 250 mg BA/kg bwt; Group 3 (BA2) rats were orally administered 500 mg BA/kg bwt; Group 4 (FPN) rats were orally administered 20 mg FPN/kg bwt; Group 5 (FPN + BA1) rats were orally administered 20 mg FPN/kg bwt plus 250 mg BA/kg bwt, and Group 6 (FPN + BA2) rats were orally administered 20 mg FPN/kg bwt plus 500 mg BA/kg bwt.

**Table 6:** Incidence and severity of histopathological findings in the examined tissue of fipronil and co-treatment with boswellic acid.

Group/Lesion	Control Rats				FPN-Rats				FPN + BA_1_ Rats				FPN + BA_2_ Rats			
	−	+	++	+++	−	+	++	+++	−	+	++	+++	−	+	++	+++
a—Seminiferous tubules																
sloughing of the germinal epithelium	6	0	0	0	0	1	2	3	5	1	0	0	5	1	0	0
necrosis of tubular epithelium	6	0	0	0	0	2	3	1	6	0	0	0	6	0	0	0
giant cell formations	6	0	0	0	1	1	2	2	6	0	0	0	6	0	0	0
hyalinization of the luminal contents	6	0	0	0	1	2	2	1	6	0	0	0	6	0	0	0
shrunken, buckled and disorganized	5	1	0	0	0	2	3	1	5	1	0	0	5	1	0	0
Atrophied tubules	6	0	0	0	1	1	2	2	6	0	0	0	6	0	0	0
b—Interstitial tissue																
inflammatory cell infiltration	6	0	0	0	2	3	1	0	6	0	0	0	6	0	0	0
hyperplasia endocrine cells	6	0	0	0	2	3	1	0	6	0	0	0	6	0	0	0
congestion of blood vessels	5	1	0	0	0	1	3	2	5	1	0	0	5	1	0	0
c—Epididymis																
sloughing of germinal epithelial cells	6	0	0	0	1	3	2	0	6	0	0	0	6	0	0	0
vacuolation of germinal epithelial cells	6	0	0	0	2	3	1	0	6	0	0	0	6	0	0	0
interstitial congestion of blood vessel	4	2	0	0	1	1	3	1	5	1	0	0	5	1	0	0
interstitial inflammatory cell infiltrations	6	0	0	0	1	1	2	2	6	0	0	0	6	0	0	0
perivascular inflammatory cell infiltrations	6	0	0	0	1	2	2	1	6	0	0	0	6	0	0	0
sperm density	6	0	0	0	0	2	2	2	6	0	0	0	6	0	0	0
d—Prostate gland																
interstitial congestion	4	2	0	0	0	1	3	2	5	1	0	0	5	1	0	0
perivascular inflammatory cell infiltration	6	0	0	0	1	1	2	2	6	0	0	0	6	0	0	0
low luminal secretions	6	0	0	0	1	1	1	3	6	0	0	0	6	0	0	0
desquamation of glandular epithelium	5	1	0	0	1	1	2	2	6	0	0	0	6	0	0	0
interstitial leukocytes infiltration	6	0	0	0	1	1	1	3	6	0	0	0	6	0	0	0
necrosis of glandular acini	5	1	0	0	1	1	2	2	6	0	0	0	6	0	0	0
e—Seminal vesicle																
leukocytes infiltration	6	0	0	0	1	1	1	3	6	0	0	0	6	0	0	0
congestion of blood vessel	5	1	0	0	0	2	2	2	5	1	0	0	5	1	0	0
necrotic tubuloalveolar glandular epithelial cells	6	0	0	0	1	2	2	1	6	0	0	0	6	0	0	0
low luminal secretions	6	0	0	0	2	2	2	0	6	0	0	0	6	0	0	0

Incidence is the number of rats with lesions per total examined. Severity of lesions was scored and graded by determining the percentage of tissue involvement. Lesion scoring: (−) absence of the lesion = 0%, (+) mild = 0–25%, (++) moderate = 25–50%, and (+++) severe = 50–100% of the examined tissue sections. FPN: fipronil; BA: boswellic acid.

## Data Availability

Not applicable.
